# Supplementary material for: SpatialData: an open and universal data framework for spatial omics
Source: Nat Methods. 2024 Mar 20;22(1):58–62. doi: 10.1038/s41592-024-02212-x (PMC11725494; doi:10.1038/s41592-024-02212-x)
Supplement: Supplementary file 1 — Supplementary Tables 1–4, Figs. 1–3 and Notes 1–5. [file 41592_2024_2212_MOESM1_ESM.pdf]

nature methods

Brief Communication

https://doi.org/10.1038/s41592-024-02212-x

SpatialData: an open and universal data framework for spatial omics

In the format provided by the authors and unedited

Supplementary tables

Table S1 | Comparison between SpatialData and SpatialFeatureExperiment on how elements that constitute a spatial omics dataset are stored and represented in memory.

Description	SpatialData	SpatialFeatureExperiment
Raster images	Image	ImgData
Raster multiscale images	Image	-
Raster labels (segmentation masks)	Labels	annotGeometries
Raster multiscale labels (segmentation masks)	Labels	-
Polygons	Shapes	colGeometries
Points (e.g. transcripts)	Points	rowGeometries
Graphs	Adata.obsp	colGraphs
Table annotations	Adata.X, Adata.var, Adata.obs	assays, rowData, colData

Table S2 | SpatialData provides a growing collection of sample datasets in the SpatialData storage format. Datasets showcasing how the data generated by various spatial profiling technologies is stored in the SpatialData on-disk format. We are continuing to add more datasets as they become available. For an up to date list, please see the online documentation: https://spatialdata.scverse.org/en/latest/tutorials/notebooks/datasets/README.html

	Number of samples/sections	File size	Description
NanoString CosMx	30 adjacent slides from the same sample	~4.2 GB	Non-small cell lung cancer (NSCLC) profiled with the Nanostring Cosmx technology[1].
10x Genomics Xenium	2 samples with overlapping area	~14.5 GB	Breast cancer tissue profile using the 10x Genomics Xenium technology[2].
10x Genomics Visium	1 sample	~1.8 GB	Breast cancer tissue profiled using the 10x Genomics Visium technology, and for the same field of view of the Xenium example[2].
CyCIF (MCMICRO output)	1 sample	~250 MB	Small lung adenocarcinoma specimen taken from a larger tissue microarray (TMA), imaged using CyCIF with three cycles and processed using MCMICRO[3].
MERFISH (Allen Institute prototype pipeline)	1 sample	~50 MB	Mouse brain section profiled with a prototype pipeline of the MERFISH technology[4].
MIBI-TOF	3 samples, 1 slide each	~25 MB	Preprocessed MIBI-TOF dataset[5] of human colorectal carcinoma.
Imaging Mass Cytometry (Steinbock output)	4 patients, total of 14 slides	~820 MB	IMC data from different patients and cancer types, processed using the Steinbock pipeline[6]. Patient 1: SCCHN (head and neck cancer)<br>Patient 2: BCC (breast cancer)<br>Patient 3: NSCLC<br>Patient 4: CRC[7,8]

Table S3 | The SpatialData library provides reader functions for common spatial omics technologies and vendor-specific file formats. Shown are technologies, associated reader function in the SpatialData library and the set of SpatialData elements used to represent the data.

Vendor/Technology	Reader function	Data	SpatialData elements
NanoString CosMx	cosmx	Transcripts locations	Points
		Raster Images	Images
		Segmentation masks	Labels
		Gene expression	Table
		Fluorescent marker intensity	Table
		Metadata	Table
10x Genomics Xenium	xenium	Transcripts locations	Points
		Raster Images	Images
		Cell segmentation	Shapes
		Nuclei Segmentation	Shapes
		Gene expression	Table
		Metadata	Table
10x Genomics Visium	visium	Raster Images	Images
		Circular regions	Shapes
		Gene expression	Table
		Metadata	Table
CyCIF (MCMICRO output)	mcmicro	Raster Images	Images
		Segmentation masks	Labels
		Protein expression	Table
		Metadata	Table
Imaging Mass Cytometry (Steinbock output)	steinbock	Raster Images	Images
		Segmentation masks	Labels
		Protein expression	Table
		Metadata	Table

Table S4 | Publicly available test datasets for verifying that readers are compliant with the SpatialData format specification. We are continuing to refine the test datasets as we receive feedback, to facilitate developers in reading data stored using the SpatialData storage format. For an up to date list, please consult the online documentation: https://spatialdata.scverse.org/en/latest/tutorials/notebooks/datasets/README.html, under "Additional resources for methods developers".

Dataset	Scope
multiple_elements.zarr	Test a SpatialData object with multiple elements: 2D single-scale and multi-scale raster types (Image, Labels); 2D vector geometries (Points, Shapes: circle, polygons, multipolygons); Table annotating the 2D Labels.
transformation_identity.zarr	Test an Identity transformation on a 2D raster type (Image) and a 2D vector type (Points).
transformation_scale.zarr	Test a Scale transformation on a 2D raster type (Image) and a 2D vector type (Points).
transformation_translation.zarr	Test a Translation transformation on a 2D raster type (Image) and a 2D vector type (Points).
transformation_affine.zarr	Test an Affine transformation on a 2D raster type (Image) and a 2D vector type (Points).
transformation_sequence.zarr	Test a Sequence transformation on a 2D raster type (Image) and a 2D vector type (Points).

Supplementary Figures

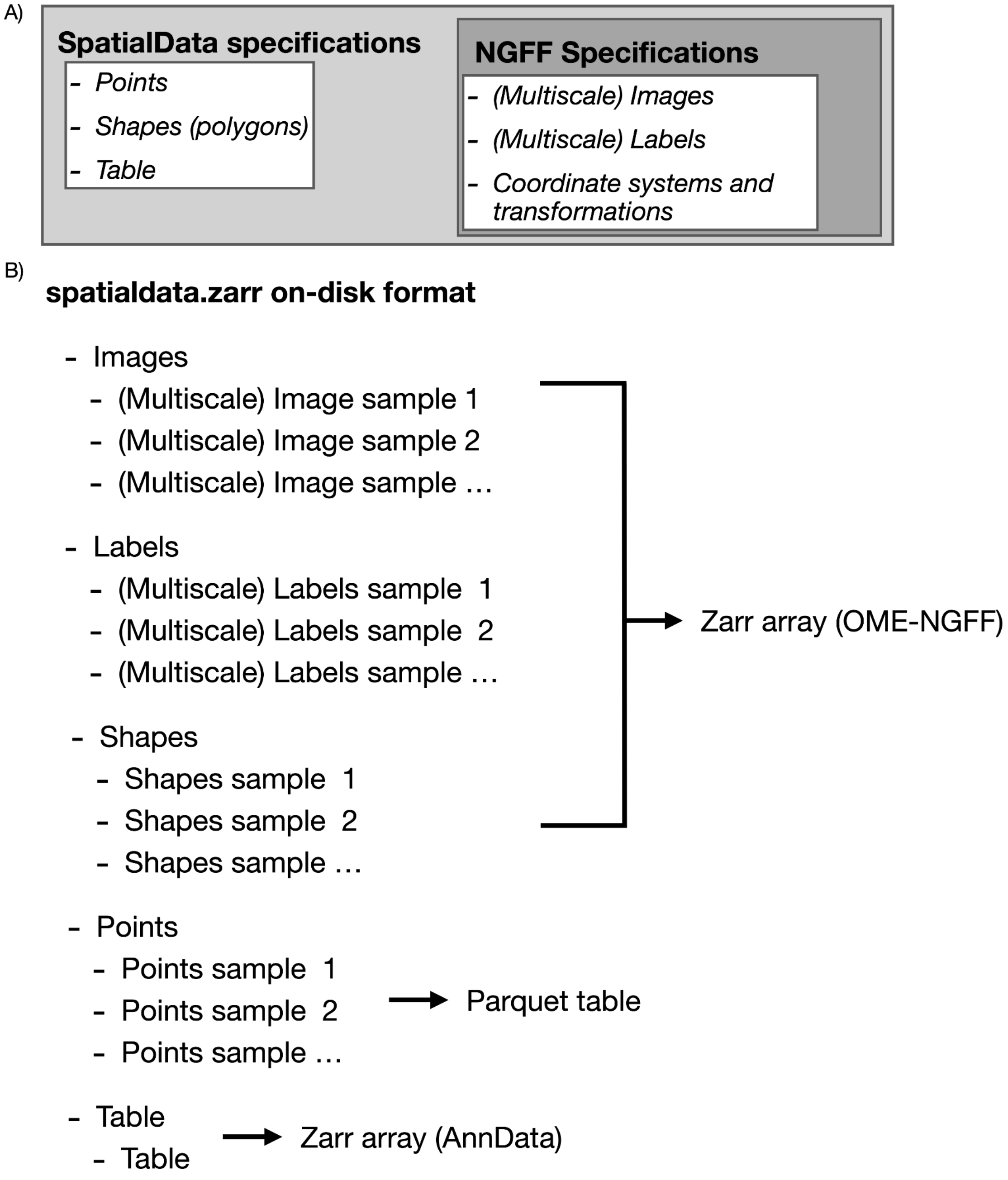

Supplementary Figure 1 | Schematic of the SpatialData storage layout in OME-Zarr. (A) The SpatialData storage format builds on the OME-NGFF specification, which we have extended by new data types required to store spatial omics information. Coordinate systems and transforms as well as tables are currently in review by the community. Points and shapes will be submitted to the community for review in a next phase to ensure interoperability. **(B)** Storage format of SpatialData: it consists of one Zarr container with nested folder structure, one for each SpatialData element. Each of the elements is saved as Zarr arrays, except for the Points, which are stored as an Apache Parquet file. We want to highlight that the Parquet file storage for points might change in the near future when a Zarr alternative will be implemented.

```
sq.gr.nhood_enrichment(sdata.table, cluster_key="leiden")
sq.pl.nhood_enrichment(sdata.table, cluster_key="leiden", figsize=(5, 5))
```

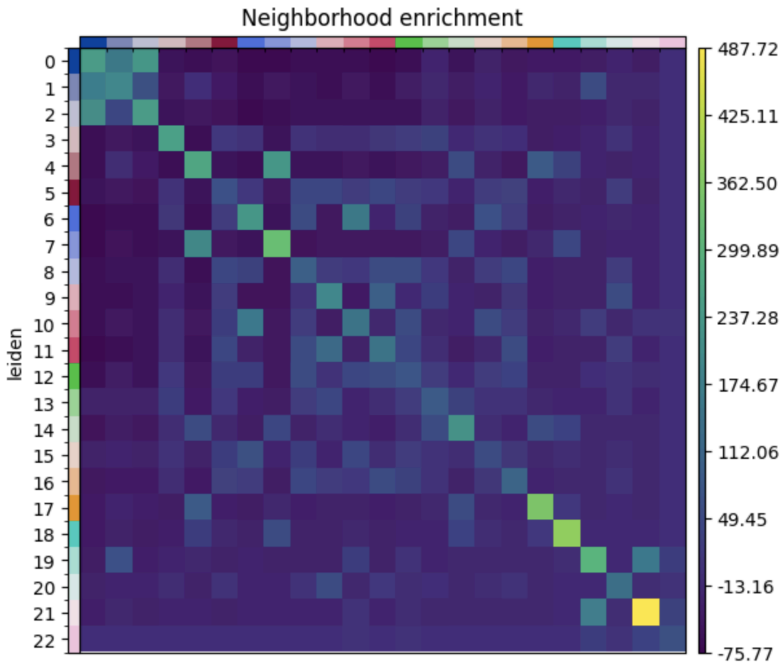

Supplementary Figure 2 | Integration of SpatialData with squidpy. SpatialData is compatible with the scverse ecosystem [9]. For example, a spatialdata can be integrated with squidpy, to compute various types of spatial summary statistics. Shown is the result from a spatial neighborhood enrichment analysis on a 10x Genomics Xenium dataset. The rows and columns of the heatmap correspond to a cluster identified in the dataset, and each entry in the heatmap represent the enrichment score: a high enrichment score means that the two clusters are found to be enriched in spatial coordinates, i.e., they are neighbors, while a low enrichment score means that the two cluster are not found to be neighbors across the tissue. See the "squidpy integration" example notebook in the online notebook for details (https://spatialdata.scverse.org/en/latest/tutorials/notebooks/notebooks/examples/squidpy_integration.html).

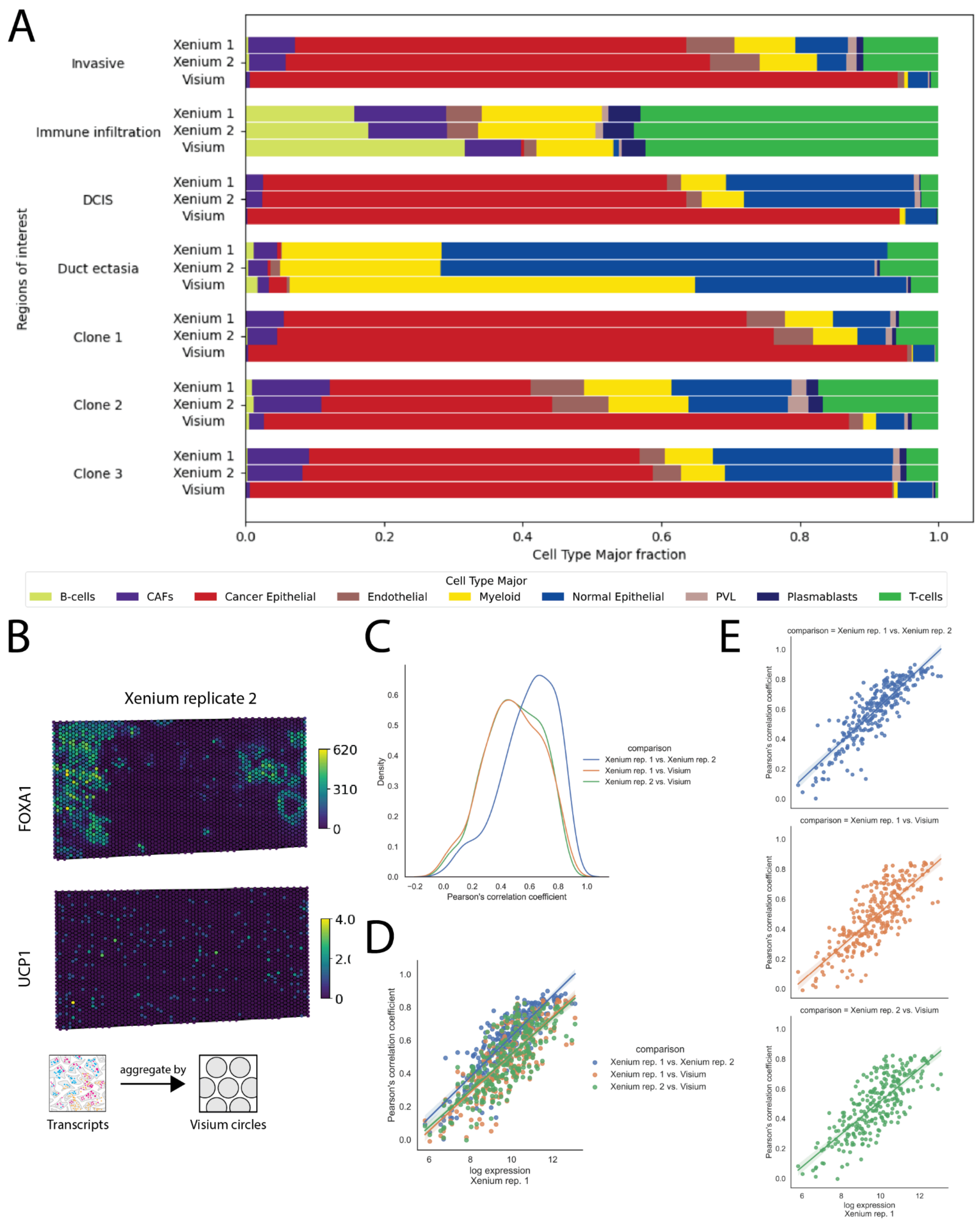

Supplementary Figure 3 | Supplementary results for the analysis presented in main text Figure 2. **(A)** Supplement of Figure 2D for the remaining ROIs and clones. Cell type proportions are computed over annotated regions of interests (ROIs) as well as clones across the Xenium replicates as well as the Visium dataset. For Visium the inferred cell type proportions using cell2location is shown. ROIs are selected based on histological features via the napari-spatialdata plug-in on the Visium-associated H&E image. The clones are inferred on the Visium data using CopyKat [10]. **(B)** Supplement of Figure 2E for replicate 2. Aggregated transcripts for *FOXA1* and *UCP1* for Xenium replicate 2 over the Visium locations. **(C)** Supplement of Figure 2E. Overall density of Pearson's correlation coefficient via pairs of sections is

computed for each gene. **(D, E)** The relationship between the Pearson's correlation coefficient between pairs of sections and overall gene expression is shown. Each point represents a gene.

Supplementary Notes

Supplementary Note 1: Challenges in spatial multi-omics data processing

Integration of spatial omics technologies for holistic views of tissue architecture

Spatial omics technologies are being applied across virtually all fields of life science research, ranging from basic biological questions in model systems[11,12], the study of disease states in human patient cohorts[13,14], to early clinical applications[15]. Current spatial omics technologies differ by readout (e.g., transcriptome, proteome, morphology), resolution and comprehensiveness[16]. For example, Visium offers whole-transcriptome readouts at the cost of spatial resolution: each Visium circular capture location describes the aggregated expression of up to dozens of cells[17]. On the other hand, single-molecule hybridization and in-situ sequencing technologies are capable of resolving millions of transcripts with diffraction-limited subcellular resolution, at the cost of the number of genes in the panel target (usually up to a few hundreds, at most low thousands)[18,19]. Increasingly, multiple technologies and profiling approaches are applied to the same samples to combine complementary information[20]. However, these efforts are stymied by a lack of methods to integrate and jointly analyze different modalities[21,22].

Existing infrastructures for managing and processing spatial omics data

Arising from the history of open source genomics and transcriptomics tools, multiple dedicated data frameworks for the analysis of spatial omics data have been proposed[16]. Typically, these data frameworks combine a storage format with a software library for accessing and processing the data. Implemented in R, SpatialExperiment[23] provides a data representation for spatial omics data embedded in the Bioconductor ecosystem[24,25]. Giotto[26] is a toolbox implemented in R for analyzing spatial omics data including algorithms for discovering spatial patterns and cell-cell communication. The in-development Giotto Suite contains a tool for interactive annotation. Squidpy[27] is a Python package embedded in the scverse ecosystem that provides both analysis algorithms and a viewer for interactive exploration. More recently, Voyager[28] provides a diverse collection of geospatial statistics and static plotting methods. All of these frameworks can be used to load data from commercial spatial omics formats and provide foundational functionality for analyzing unimodal molecular profiles. However, at present the storage formats and associated libraries are primarily tailored to the processing and exploration of unimodal molecular profiles and lack the infrastructure for the analysis of large image data, perform multimodal spatial alignment, and derive interactive annotation (c.f. Extended Data Table 1).

In a complementary effort to developing data frameworks, researchers have created databases to aggregate spatial omics datasets. Databases are useful as they provide a centralized location to find existing datasets. In part these efforts also provide a uniform interface for processing data by returning the data in a standardized format. Earlier databases such as SpatialDB[29] focused on storing data and provided limited infrastructure for exploring the data. Other efforts such as SOAR[30] and STOmicsDB[31] provide richer interactive exploration and analysis capabilities such as finding spatially variable genes or cell-cell interactions via the web interface.

However, these tools do not provide programmatic access to the data and analysis results and thus are difficult to integrate into analysis pipelines. Recently, SODB[32] was released and provides many datasets in a uniform format based on the squidpy AnnData[27] layout and data exploration capabilities via the web interface (e.g., finding molecular markers from tissue regions). SODB also provides a Python interface for programmatic access to the database. Notably, SODB relies on existing data storage solutions that are primarily designed for conventional single-cell omics, and the software is not available in standard packaging indexes (e.g., PyPI or conda-forge), thus limiting its use as a foundational data infrastructure for more specialized software to build on. More generally, all current database solutions provide datasets using existing formats and frameworks. Therefore, while essential for improving the findability of data, these efforts do not address the need for foundational solutions for data integration and processing.

Limitations of existing spatial omics data processing solutions

While the functionalities offered by SpatialData are at least in part also provided by existing solutions (Extended Data Table 1), we have identified a set of four key requirements that are uniquely addressed by SpatialData:

- Support for large-scale image data
- Spatial alignment of multimodal spatial omics data
- Cross-modality aggregation
- Interactive spatial annotation

Recognizing the valuable functionality provided by the existing ecosystem of tools, we have prioritized interoperability in the design of SpatialData so that the infrastructure can both extend the capabilities of existing software and serve as a foundation upon which new tools can be built.

Integration of large image data

Access to image data provides important complementary information to aggregated molecular profiles. For example, H&E images provide histological and anatomical context for molecular measurements. Multidimensional images are generally stored as dense arrays, which can be 10s-100s GB in size. Owing to their size, images require special consideration for performant processing and integration with molecular profiles. For example, lazy loading and multiscale representations only load the required portions and detail level of an image required for a given processing operation and thus allow images larger than the available memory to be processed. As a result, image processing software has largely been siloed from molecular profile analysis software. Thus, there is the need for a spatial omics analysis framework that fully embraces image data but at the same time facilitates the seamless integration with molecular features.

Alignment of multimodal spatial omics data

Each spatial omics modality measures specific features of molecular architecture (e.g., RNA expression, morphology, chromatin accessibility). To build a holistic understanding of tissue architecture, it is important to be able to combine complementary modalities and transfer

quantifications and annotations between them. For example, if two regions of interest are identified by histological features in an H&E image, we may want to aggregate and compare the molecular profiles of protein abundance and RNA expression in those ROIs to study the signaling that gives rise to the distinct histological features. In order to achieve such an integration, disparate datasets need to be aligned into a common coordinate system (CCS). Computationally, this requires functionality to spatially transform all data types, which is non-trivial, as there are many types of data in spatial omics datasets (e.g., multidimensional arrays, polygons, points). Further, multiple aligned coordinate spaces may be required to represent the multiscale nature of biological architecture (e.g., an organ-specific coordinate system, and a tissue-specific coordinate system). Thus, to achieve practical multimodal spatial alignment, a flexible transformation system that supports multiple coordinate systems is required.

Cross-modality integration

In addition to aligning multiple datasets, it is necessary to transfer spatial annotations between datasets in order to efficiently leverage complementary insights that are provided by each modality. In the context of the example above, transferring the spatial annotations of ROIs derived from the H&E image to the molecular profiles enables stratification and comparison of the signaling in distinct biological compartments. Such a comparison would not have been possible without transferring the annotations from the H&E image to the molecular profiles. While conceptually straight-forward, transferring spatial annotations across modalities is non-trivial to achieve in practice, as the corresponding aggregation operations must be applied to different data types (e.g., multidimensional arrays, polygons, points). Thus, to achieve cross-modality integration, a uniform interface for aggregating all data types present in spatial omics datasets is indispensable.

Interactive annotation

Interpreting spatial omics datasets requires input from domain experts. For example, it is often desirable to annotate regions of interest based on histological or anatomical features. Such annotations are necessary to compare distinct anatomical compartments and provide ground truth for training and validating analysis algorithms. Owing to the size and heterogeneity of spatial omics data types, interactive analysis of spatial omics datasets requires a performant viewer that can represent diverse data types and is seamlessly integrated with the underlying storage solution.

Comparisons of storage and objects for spatial omics data handling

We evaluated the libraries in Extended Data Table 1 on the basis described below.

Data types

Data types include the type of representations that constitute a building block of the spatial omics experiment:

- Raster images: multiplexed microscopy images.
- Raster labels: segmentation masks.

- Multiscale raster: pyramid-like representation of large microscopy images or labels. This is important for performant rendering and access of large images.
- Polygons: list of polygons representing regions of interest (e.g. pathology annotation, tissue regions)
- Regular shapes: similar to polygons, used to represent capture locations of array-based technologies (e.g. circles for Visium, squares for DBiT-seq etc.)
- Points: list of annotated points, used to represent e.g. transcripts locations.
- Features matrix: gene or protein expression matrix.
- Annotation matrix: experiment metadata, cluster annotation etc.
- Graphs: neighbors graphs between regions (cells, spots, shapes) or features (genes, proteins).

Operations

These include operations to process spatial omics experiments, such as obtaining crops or slices of the data, summary statistics and data type conversion.

- Points aggregation: compute summary statistics between points and regions (labels, polygons or shapes) such as counting the number of transcripts across segmented cells.
- Geometry intersection: set operations between polygons, labels or shapes.
- Transforms: transform elements (images, regions and points) between coordinate systems
- Coordinate systems: support for specifying different coordinate systems for each element (e.g. pixel-based coordinate systems versus global physical coordinate systems).

Plotting

Plotting can be static or interactive.

Supplementary Note 2: Universal representation of spatial omics data

Spatial omics datasets can comprise a variety of different data types. The SpatialData storage format and the corresponding in-memory representation support five primitive SpatialElements to represent different datasets and data types: Images, Labels, Points, Shapes, and Tables (explained below in detail). These SpatialElements allow for representing raw and derived data for a wide range of spatial omics assays, and we provide convenient reader functions for common spatial omics data formats (Table S3). Multiple SpatialElements can be grouped together within a SpatialData object. SpatialData objects are stored on disk in the SpatialData format, which is built upon the Zarr implementation of the OME-NGFF [33,34] specification (Supplementary Figure 1). Briefly, OME-NGFF is a community-driven data standard with readers in Python, Java, and JavaScript, enhancing the interoperability of the SpatialData storage format. Using the standardized metadata from OME-NGFF further improves the accessibility and reproducibility of SpatialData.

- **Images**: images are raster data that store high-resolution microscopy images. They are stored as Zarr arrays and are represented in-memory as a (multiscale) *SpatialImage* class[35]. *SpatialImage* inherits from *xarray*[36] and *xarray-datatree*[37] for representing and manipulating high-dimensional arrays with named coordinates.
- **Labels**: labels are raster data that contain regions of interest such as segmentation masks. They are stored similarly to images as Zarr arrays on disk and represented in-memory as (multiscale) *SpatialImage*.
- **Shapes**: shapes are polygon data that contain regions of interest such as cell segmentations, capture locations of array-based spatial transcriptomics data or other types of ROIs. They are stored as a series of arrays that contain coordinates and offsets of the polygons as Zarr arrays on disk and represented in-memory as Shapely[38] objects in GeoPandas[39] dataframes.
- **Points**: points contain large collections (typically order of millions or billion) of coordinates and annotations such as transcripts locations and their associated metadata. They are stored as a parquet file on disk and represented in-memory as a lazy object with a DaskDataFrame[40].
- **Tables**: tables store molecular profile information (gene expression, protein expression etc.) and associated metadata for observations and variables. It also stores the adjacency matrix of spatial graphs as well as any relevant additional metadata. It is stored on disk and represented in memory as AnnData[41].

To illustrate how the SpatialData format works for common spatial omics assays, we have converted 42 fields of view from 7 different technologies in the SpatialData format and made them available as example datasets (Table S2). These standardized datasets will be extended and available online, providing methods developers a starting point to benchmark new computational approaches across different spatial omics technologies and modalities.

Supplementary Note 3: Scalability of image storage in SpatialData

To ensure scalability to large datasets, SpatialData relies on the next-generation file format, OME-Zarr, which has been specifically designed for processing and visualization of large scientific data [33,34]. The raw image data tends to comprise the largest portion of a (processed) spatial omics dataset, thus is the key data storage bottleneck. In particular for large file sizes, OME-Zarr can be over 10 times faster than traditional image formats such as TIFF when accessing from local storage and over 100 times faster when accessing data from cloud storage [34].

Briefly, OME-Zarr implements chunked storage of binary, compressed data to support performant parallel writing and reading of large image data. Consequently, OME-Zarr scales to datasets of hundreds of terabytes in size [33], either stored as individual image volumes or collections of images. Additionally, OME-Zarr supports a multiscale representation of each image (i.e. image pyramid) to enable seamless zooming during interactive viewing of high resolution images and segmentation masks. Through this underlying infrastructure, SpatialData can load data lazily, permitting pipelines utilizing SpatialData to efficiently load and process large datasets.

We note that in practice the performance and scalability of processing large datasets will critically depend on the underlying data storage and computation infrastructure. In particular, the ability to lazily load data in parallel is closely tied to the chunk configuration of Zarr stores. In practice, this can lead to a large number of files per image. Object storage such as S3 is often the preferred file system for handling large file numbers. Additionally, the next major version of Zarr, v3, will support “sharding” which reduces the number of files by storing multiple chunks in a single file [42].

Supplementary Note 4: Training of deep learning models directly from SpatialData datasets.

Algorithms based on deep learning have shown great promise for integration and prediction tasks on spatial data. Training such models requires careful curation of training datasets. Dataset preparation is especially time consuming for spatial omics data due to heterogeneous data types, dataset sizes, and the need for spatially-aligning multiple modalities. Leveraging the spatial query API, SpatialData comes with data loaders that are derived from the PyTorch Dataset class[43], thereby facilitating data ingestion for deep learning applications (Figure 1D, Extended Data Figure 5). The Dataset implementation uses the spatial query functionality to generate tiles from a SpatialData object. This implementation enables users to integrate SpatialData datasets with the rich Python deep learning ecosystem including models and infrastructure from MONAI.

A tutorial on how to use the PyTorch dataset loader is available as part of the online documentation (https://spatialdata.scverse.org/en/latest/tutorials/notebooks/notebooks/examples/densenet.html), which illustrates the use of the SpatialData PyTorch dataset interface to train a MONAI DenseNet encoder on the breast cancer study as in the main text Figure 2B. The tutorial considers the generation of image tiles from an H&E image that is spatially aligned to one of the two Xenium datasets, by querying the image tiles around each Xenium cell using the SpatialData PyTorch Dataset class. We then use these data to train DenseNet to predict the cell type from each image tile.

Supplementary Note 5: Reference datasets for facilitating interoperability.

To support interoperability and method development efforts, we have implemented the SpatialData format using storage technologies with broad reader support. In particular, we have targeted JavaScript as it is well-suited for web viewers and R/Bioconductor because it is one of the most popular languages for bioinformatics. Leveraging the implementation of SpatialData in Zarr and Parquet, SpatialData files can be accessed in JavaScript and R using existing readers. For example, Zarr raster data can be read using: zarr.js[44] (JavaScript) and Rarr[45] (R), and Parquet vector geometries can be read using parquet.js (JavaScript) and arrow (R). Currently, tabular data stored in Zarr following the AnnData specification can be read in JavaScript (see https://anndata.readthedocs.io/en/latest/interoperability.html), and ongoing external work is being carried out to extend AnnDataR to support Zarr files (see https://github.com/scverse/anndataR/issues/91).

To make it seamless for users to load SpatialData files in JavaScript and R, we aim to support the work of the developers of spatial omics projects in those communities (e.g., Vitessce[46], SpatialFeatureExperiment[28], SpatialExperiment[23]) in developing specific readers for the SpatialData storage format. To support the development of readers in other programming languages, we have created test datasets that provide examples of the core parts of the specification and made them publicly available (Table S4). Developers can use these test datasets to efficiently verify that their readers are compliant with the SpatialData specification. Furthermore, developers can also test their methods with the example datasets from existing studies that we converted and made available in the cloud. The description of these datasets is given in the Table S2, and they can be accessed from the SpatialData online documentation.

Supplementary Note 6: Limitations and future directions.

The development of the SpatialData framework is an ongoing collaborative effort carried by the scVerse open source community. As such, we encourage feedback and contributions from the users in order to continuously improve the robustness, scalability, interoperability and generality of the framework.

On the topic of scalability, as data from new technologies continues to grow in size, we are focusing on improving the performance when performing spatial operations (query, aggregation, tiling) and during static and interactive visualization of large collections of geometries. We are also exploring a multiscale representation for vector data (points, polygons).

On the topic of interoperability and generality, the SpatialData framework already supports the storage and manipulation of 3D raster data (images, labels) and 3D points, but polygons are currently constrained to have 2 dimensions. Full 3D support in SpatialData will require the ability to store 3D vector objects (e.g., polygonal meshes). This will enable the full generalization of the various operations, in particular spatial queries and aggregation, for 3 dimensional objects. In addition, we are exploring the support of a time axis in order to be able to store 5D volumetric images and time series of geometries.

References

1. He, S. *et al.* High-Plex Multiomic Analysis in FFPE Tissue at Single-Cellular and Subcellular Resolution by Spatial Molecular Imaging. *bioRxiv* 2021.11.03.467020 (2021) doi:10.1101/2021.11.03.467020.
2. Janesick, A. *et al.* High resolution mapping of the breast cancer tumor microenvironment using integrated single cell, spatial and in situ analysis of FFPE tissue. *bioRxiv* 2022.10.06.510405 (2022) doi:10.1101/2022.10.06.510405.
3. Schapiro, D. *et al.* MCMICRO: A scalable, modular image-processing pipeline for multiplexed tissue imaging. *Cold Spring Harbor Laboratory* 2021.03.15.435473 (2021) doi:10.1101/2021.03.15.435473.
4. Moffitt, J. R. *et al.* Molecular, spatial, and functional single-cell profiling of the hypothalamic preoptic region. *Science* **362**, (2018).
5. Hartmann, F. J. *et al.* Single-cell metabolic profiling of human cytotoxic T cells. *Nat. Biotechnol.* (2020) doi:10.1038/s41587-020-0651-8.
6. Windhager, J., Bodenmiller, B. & Eling, N. An end-to-end workflow for multiplexed image processing and analysis. *bioRxiv* 2021.11.12.468357 (2021) doi:10.1101/2021.11.12.468357.
7. Eling, N. & Windhager, J. *Example imaging mass cytometry raw data*. (2022). doi:10.5281/zenodo.5949116.
8. Eling, N. & Windhager, J. *steinbock results of IMC example data*. (2022). doi:10.5281/zenodo.7412972.
9. Virshup, I. *et al.* The scverse project provides a computational ecosystem for single-cell omics data analysis. *Nat. Biotechnol.* **41**, 604–606 (2023).
10. Gao, R. *et al.* Delineating copy number and clonal substructure in human tumors from single-cell transcriptomes. *Nat. Biotechnol.* **39**, 599–608 (2021).

11. Lohoff, T. *et al.* Integration of spatial and single-cell transcriptomic data elucidates mouse organogenesis. *Nat. Biotechnol.* (2021) doi:10.1038/s41587-021-01006-2.

12. van den Brink, S. C. *et al.* Single-cell and spatial transcriptomics reveal somitogenesis in gastruloids. *Nature* **582**, 405–409 (2020).

13. Jackson, H. W. *et al.* The single-cell pathology landscape of breast cancer. *Nature* **578**, 615–620 (2020).

14. Lin, J.-R. *et al.* Multiplexed 3D atlas of state transitions and immune interaction in colorectal cancer. *Cell* **186**, 363–381.e19 (2023).

15. Irmisch, A. *et al.* The Tumor Profiler Study: integrated, multi-omic, functional tumor profiling for clinical decision support. *Cancer Cell* **39**, 288–293 (2021).

16. Moses, L. & Pachter, L. Museum of spatial transcriptomics. *Nat. Methods* **19**, 534–546 (2022).

17. Salmén, F. *et al.* Barcoded solid-phase RNA capture for Spatial Transcriptomics profiling in mammalian tissue sections. *Nat. Protoc.* **13**, 2501–2534 (2018).

18. Eng, C.-H. L. *et al.* Transcriptome-scale super-resolved imaging in tissues by RNA seqFISH. *Nature* **568**, 235–239 (2019).

19. Chen, K. H., Boettiger, A. N., Moffitt, J. R., Wang, S. & Zhuang, X. RNA imaging. Spatially resolved, highly multiplexed RNA profiling in single cells. *Science* **348**, aaa6090 (2015).

20. He, S. *et al.* High-plex imaging of RNA and proteins at subcellular resolution in fixed tissue by spatial molecular imaging. *Nat. Biotechnol.* **40**, 1794–1806 (2022).

21. Rao, A., Barkley, D., França, G. S. & Yanai, I. Exploring tissue architecture using spatial transcriptomics. *Nature* **596**, 211–220 (2021).

22. Palla, G., Fischer, D. S., Regev, A. & Theis, F. J. Spatial components of molecular tissue biology. *Nat. Biotechnol.* (2022) doi:10.1038/s41587-021-01182-1.

23. Righelli, D. *et al.* SpatialExperiment: infrastructure for spatially resolved transcriptomics data in R using Bioconductor. *Cold Spring Harbor Laboratory* 2021.01.27.428431 (2021)

doi:10.1101/2021.01.27.428431.

24. Gentleman, R. C. *et al.* Bioconductor: open software development for computational biology and bioinformatics. *Genome Biol.* **5**, R80 (2004).
25. Huber, W. *et al.* Orchestrating high-throughput genomic analysis with Bioconductor. *Nat. Methods* **12**, 115–121 (2015).
26. Dries, R. *et al.* Giotto: a toolbox for integrative analysis and visualization of spatial expression data. *Genome Biol.* **22**, 78 (2021).
27. Palla, G. *et al.* Squidpy: a scalable framework for spatial omics analysis. *Nat. Methods* **19**, 171–178 (2022).
28. Moses, L., Jackson, K., Luebbert, L. & Pachter, L. Voyager: From geospatial to spatial omics. Preprint at https://github.com/pachterlab/voyager (2023).
29. Fan, Z., Chen, R. & Chen, X. SpatialDB: a database for spatially resolved transcriptomes. *Nucleic Acids Res.* **48**, D233–D237 (2020).
30. Li, Y. *et al.* Spatial transcriptomics resources to model spatial variability and cell-cell interactions. *bioRxiv* 2022.04.17.488596 (2022) doi:10.1101/2022.04.17.488596.
31. Xu, Z. *et al.* STOmicsDB: a database of Spatial Transcriptomic data. *bioRxiv* 2022.03.11.481421 (2022) doi:10.1101/2022.03.11.481421.
32. Yuan, Z. *et al.* SODB facilitates comprehensive exploration of spatial omics data. *Nat. Methods* **20**, 387–399 (2023).
33. Moore, J. *et al.* OME-Zarr: a cloud-optimized bioimaging file format with international community support. *bioRxiv* 2023.02.17.528834 (2023) doi:10.1101/2023.02.17.528834.
34. Moore, J. *et al.* OME-NGFF: a next-generation file format for expanding bioimaging data-access strategies. *Nat. Methods* **18**, 1496–1498 (2021).
35. McCormick, M. *spatial-image/spatial-image: spatial-image 0.2.1*. (2022). doi:10.5281/zenodo.6508869.
36. Hoyer, S. & Hamman, J. J. xarray: N-D labeled Arrays and Datasets in Python. *J. Open*

Res. Softw. **5**, (2017).

37. *datatree: WIP implementation of a tree-like hierarchical data structure for xarray*. (Github).
38. Gillies, S. *et al. Shapely*. (Zenodo, 2023). doi:10.5281/ZENODO.5597138.
39. Jordahl, K. *et al. geopandas/geopandas: v0.8.1*. (2020). doi:10.5281/zenodo.3946761.
40. Dask Development Team. Dask: Library for dynamic task scheduling. Preprint at https://dask.org (2016).
41. Virshup, I., Rybakov, S., Theis, F. J., Angerer, P. & Alexander Wolf, F. anndata: Annotated data. *bioRxiv* 2021.12.16.473007 (2021) doi:10.1101/2021.12.16.473007.
42. Zarr core specification (version 3.0) — Zarr specs documentation. https://zarr-specs.readthedocs.io/en/latest/v3/core/v3.0.html.
43. Paszke, A. *et al.* Automatic Differentiation in PyTorch. in *NIPS Autodiff Workshop* (2017).
44. Zuidhof, G. *zarr.js: Javascript implementation of Zarr*. (Github).
45. Rarr. *Bioconductor* https://bioconductor.org/packages/release/bioc/html/Rarr.html.
46. Keller, M. S. *et al.* Vitessce: a framework for integrative visualization of multi-modal and spatially-resolved single-cell data. Preprint at https://doi.org/10.31219/osf.io/y8thv (2021).
