# Supplementary material for: SpatialData: an open and universal data framework for spatial omics
Source: Nat Methods. 2024 Mar 20;22(1):58–62. doi: 10.1038/s41592-024-02212-x (PMC11725494; doi:10.1038/s41592-024-02212-x)
Supplement: Supplementary file 2 — Reporting Summary [file 41592_2024_2212_MOESM2_ESM.pdf]

nature portfolio

Corresponding author(s): Josh Moore, Fabian J. Theis, Oliver Stegle

Last updated by author(s): Jan 3, 2024

Reporting Summary

Nature Portfolio wishes to improve the reproducibility of the work that we publish. This form provides structure for consistency and transparency in reporting. For further information on Nature Portfolio policies, see our Editorial Policies and the Editorial Policy Checklist.

Statistics

For all statistical analyses, confirm that the following items are present in the figure legend, table legend, main text, or Methods section.

n/a	Confirmed	
☒	☐	The exact sample size (*n*) for each experimental group/condition, given as a discrete number and unit of measurement
☒	☐	A statement on whether measurements were taken from distinct samples or whether the same sample was measured repeatedly
☒	☐	The statistical test(s) used AND whether they are one- or two-sided<br>*Only common tests should be described solely by name; describe more complex techniques in the Methods section.*
☒	☐	A description of all covariates tested
☒	☐	A description of any assumptions or corrections, such as tests of normality and adjustment for multiple comparisons
☒	☐	A full description of the statistical parameters including central tendency (e.g. means) or other basic estimates (e.g. regression coefficient) AND variation (e.g. standard deviation) or associated estimates of uncertainty (e.g. confidence intervals)
☒	☐	For null hypothesis testing, the test statistic (e.g. *F*, *t*, *r*) with confidence intervals, effect sizes, degrees of freedom and *P* value noted<br>*Give P values as exact values whenever suitable.*
☒	☐	For Bayesian analysis, information on the choice of priors and Markov chain Monte Carlo settings
☒	☐	For hierarchical and complex designs, identification of the appropriate level for tests and full reporting of outcomes
☐	☒	Estimates of effect sizes (e.g. Cohen's *d*, Pearson's *r*), indicating how they were calculated

Our web collection on statistics for biologists contains articles on many of the points above.

Software and code

Policy information about availability of computer code

Data collection

The data used in this study was downloaded from public sources using custom Python script that we made available at https://github.com/giovp/spatialdata-sandbox. Such scripts download the raw data and convert it to the SpatialData format. The converted data is also accessible (see Data Availability statement).

Data analysis

All code used to perform the analyses and generate figures in this manuscript is available here: https://github.com/scverse/spatialdata-notebooks/tree/main/notebooks/paper_reproducibility and has the following software requirements:
spatialdata>=0.0.15
spatialdata-io>=0.0.9
spatialdata-plot>=0.0.6
napari-spatialdata>=0.3.1
cell2location>=0.1.3
copykat>=1.1.0

The packages of the SpatialData framework (spatialdata, spatialdata-io, spatialdata-plot, napari-spatialdata) and all their dependencies can be installed automatically via pip, which is the recommended way to install the libraries (conda support is in preparation).
Here below is the list of dependent libraries and corresponding versions used at the time of writing this manuscript.
Requirements of the spatialdata package:
anndata>=0.9.1
numpy>=1.24.3
xarray>=2022.12.0
zarr>=2.14.2

ome_zarr>=0.7.0
spatial_image>=0.3.0
multiscale_spatial_image>=0.11.2
xarray-schema>=0.0.3
geopandas>=0.13.0
shapely>=2.0.1
rich>=13.3.1
pyarrow>=11.0.0
typing_extensions>=4.9.0
dask-image>=2022.9.0
networkx>=2.8.4
xarray-spatial>=0.3.5
tqdm>=4.65.0
Requirements of the spatialdata-io package:
scikit-image>=0.22.0
h5py>=3.9.0
imagecodecs>=2023.9.4
joblib>=1.3.2
readfcs>=1.1.7
Requirements of the spatialdata-plot package:
matplotlib>=3.8.2
scikit-learn>=1.3.2
scanpy>=1.9.6
matplotlib-scalebar>=0.8.1
Requirements of the napari-spatialdata package:
click>=8.1.7
cycler>=0.12.1
loguru>=0.7.2
napari>=0.4.16
napari-matplotlib>=1.2.0
numba>=0.58.1
packaging>=23.2
pillow>=10.0.0
qtpy>=2.4.1
scipy>=1.11.4
superqt>=0.6.1
vispy>=0.10.0

For manuscripts utilizing custom algorithms or software that are central to the research but not yet described in published literature, software must be made available to editors and reviewers. We strongly encourage code deposition in a community repository (e.g. GitHub). See the Nature Portfolio guidelines for submitting code & software for further information.

Data

Policy information about availability of data

All manuscripts must include a data availability statement. This statement should provide the following information, where applicable:

- Accession codes, unique identifiers, or web links for publicly available datasets
- A description of any restrictions on data availability
- For clinical datasets or third party data, please ensure that the statement adheres to our policy

We converted several example datasets to Zarr using the SpatialData package (see Software and Code). At the time of writing, we include data from the following technologies: NanoString CosMx, 10x Genomics Xenium, 10x Genomics Visium, CyCIF, MERFISH, MIBI-TOF, Imaging Mass Cytometry. The converted data is accessible from https://spatialdata.scverse.org/en/latest/tutorials/notebooks/datasets/README.html. For an overview of the datasets and their respective source publication, please refer to Table S4.

Human research participants

Policy information about studies involving human research participants and Sex and Gender in Research.

Reporting on sex and gender	No participants have been recruited for this study.
Population characteristics	No participants have been recruited for this study.
Recruitment	No participants have been recruited for this study.
Ethics oversight	No participants have been recruited for this study.

Note that full information on the approval of the study protocol must also be provided in the manuscript.

Field-specific reporting

Please select the one below that is the best fit for your research. If you are not sure, read the appropriate sections before making your selection.

☒ Life sciences ☐ Behavioural & social sciences ☐ Ecological, evolutionary & environmental sciences

For a reference copy of the document with all sections, see nature.com/documents/nr-reporting-summary-flat.pdf

Life sciences study design

All studies must disclose on these points even when the disclosure is negative.

Sample size	NanoString CosMx data: 1 sample, 30 slides.<br>10x Genomics Xenium + Visium (breast cancer, Janesick et al.): 1 sample, 3 slides<br>CyCIF MCMICRO: 1 sample, 1 slide<br>MERFISH: 1 sample, 1 slide<br>MIBI-TOF: 3 samples, 1 slide each<br>Imaging Mass Cytometry: 4 patients, 14 slides total<br>10x Genomics Visium (prostate cancer, Erikson et al.): 1 sample, 15 fields-of-view<br><br>The sample sizes and slides number were chosen to be often greater than 1 to demonstrate the integration capabilities of the framework.
Data exclusions	No data has been excluded during our data processing.
Replication	The code to generate all figures is publicly available. See Software and Data Availability section.
Randomization	We did not require randomization in the data. We did not divide the data into subgroups.
Blinding	We did not divide the data into subgroups.

Reporting for specific materials, systems and methods

We require information from authors about some types of materials, experimental systems and methods used in many studies. Here, indicate whether each material, system or method listed is relevant to your study. If you are not sure if a list item applies to your research, read the appropriate section before selecting a response.

Materials & experimental systems

n/a	Involved in the study	
☒	☐	Antibodies
☒	☐	Eukaryotic cell lines
☒	☐	Palaeontology and archaeology
☒	☐	Animals and other organisms
☒	☐	Clinical data
☒	☐	Dual use research of concern

Methods

n/a	Involved in the study	
☒	☐	ChIP-seq
☒	☐	Flow cytometry
☒	☐	MRI-based neuroimaging
